# Pyrene-POSS nanohybrid as a dispersant for carbon nanotubes in solvents of various polarities: its synthesis and application in the preparation of a composite membrane

**DOI:** 10.1186/1556-276X-7-296

**Published:** 2012-06-07

**Authors:** Shahid Majeed, Volkan Filiz, Sergey Shishatskiy, Jan Wind, Clarissa Abetz, Volker Abetz

**Affiliations:** 1 Institute of Polymer Research, Helmholtz-Zentrum Geesthacht, Max-Planck- Str.1, 21502, Geesthacht, Germany

**Keywords:** Pyrene-POSS, Carbon nanotube dispersion, Non-covalent functionalization, Conductive membranes

## Abstract

In this study we report the preparation of nanohybrid dispersant molecules based on pyrene and polyhedral oligomeric silsesquioxanes for non-covalent functionalization of multi-walled carbon nanotubes (MWCNTs). The prepared dispersant improves the dispersion of MWCNTs in organic solvents with very different polarities such as tetrahydrofuran, toluene, and *n*-hexane. The functionalized MWCNTs were used to introduce conductivity into polydimethylsiloxane membranes which can be used for electrostatic discharge applications.

## Background

The extraordinary electrical, mechanical, and thermal properties of carbon nanotubes (CNTs) make them the strongest candidates for their application in composite materials [1-3]. Good dispersion of CNTs is required for their application in many composites. However, CNTs are produced in the form of bundles where they are attracted together by van der Waals interactions. The aggregation of CNTs in the form of bundles influences the properties of the resulting composite materials e.g., ineffective stress transfer and higher percolation thresholds for electrical conductivity. Moreover, the agglomerates of carbon nanotubes may act as conventional carbon black and hence, to obtain improved material properties, the disaggregation of CNTs agglomerates is necessary [4-6].

Surface modification of CNTs is a tool to improve their dispersion in various solvents and matrices and it can be grouped into two different categories: (a) binding the functional groups on the π-conjugated skeleton of carbon nanotube via covalent bonding [7-9] and (b) physical adsorption or wrapping of a variety of functional molecules via non-covalent interactions [10-14]. Non-covalent functionalization has an advantage over covalent functionalization because no major side wall defects occur thus preserving the electronic properties of π-conjugated tubular structure of CNTs. Such functionalization involves wrapping of the CNTs’ surface by various polymers, polynuclear aromatic compounds, surfactants, or biomolecules [14]. Ionic and biological surfactants improve the CNTs’ dispersion in aqueous solutions where CNTs are entrapped into micelles leading to a stable dispersion [15]. Conjugated polymers interact with CNTs by π−π stacking, resulting in better dispersion of CNTs in specific organic solvents [16]. Block copolymers, having at least one block exhibiting conjugation and other having high affinity toward solvent, lead to a better dispersion of CNTs in solvents of different polarities [17]. Polycyclic aromatic compounds e.g., pyrene, are also well known for their π−π stacking on CNTs surface, and the attachment of pyrene to molecular species which are soluble in organic solvents or aqueous media creates the possibility to effectively disperse CNTs in these solvents [15].

Polyhedral oligomeric silsesquioxanes (POSS) possess an inorganic cage structure, with the possibility for a variety of functional groups of different nature to be attached to the Si_8_O_12_ core [18,19]. The reactive functional groups on POSS have been subjected to functionalize CNTs, allowing a good dispersion of CNTs in solvents such as chloroform and tetrahydrofuran (THF) [20-22]. POSS-functionalized CNTs resulted in nanocomposites with improved CNTs dispersion and mechanical properties [22]. In this study, 1-pyrenebutyric acid was attached covalently to POSS, and multi-walled carbon nanotubes (MWCNTs) were non-covalently modified with the resultant pyrene-POSS. The obtained hybrid material can be efficiently dispersed in various organic solvents such as *n*-hexane, toluene, and THF to form stable dispersions. The dispersibility of the hybrid material is provided by the presence of the POSS with aliphatic moieties having high affinity toward organic solvents.

To demonstrate the dispersing effect of pyrene-POSS also in a polymer matrix, pyrene-POSS-modified MWCNTs were used for the fabrication of conductive polydimethylsiloxane (PDMS) nanocomposite membranes. Such conductive membranes, when applied in spiral wound membrane modules with plastic housing, can provide the opportunity to neutralize electrostatic charges and hence may provide, for example, a safer separation of hydrocarbons*.* In order to avoid electrostatic charging, a surface resistivity between 10^6^ Ω/□ and 10^9^ Ω/□ or bulk electrical conductivity above 10^−6^ Sm^−1^ are required [23,24].

It has been a challenge to uniformly disperse non-functionalized MWCNTs in a thin PDMS selective layer because of the indispersibility of unmodified MWCNTs in nonpolar solvents like *n*-hexane and toluene. However, pyrene-POSS provided a way to effectively disperse MWCNTs in PDMS nanocomposite membranes with the advantage of a well-preserved tubular conjugated MWCNTs structure which, in turn, conserves their electrical properties. The PDMS nanocomposite membranes were characterized by optical microscopy, scanning electron microscopy, gas permeation, and sheet resistance measurements.

## Methods

### Materials

1-Pyrenebutyric acid from Aldrich (Sigma-Aldrich Logistik GmbH, Schnelldorf, Germany) and aminopropylisobutyl POSS (APiB-POSS) from Hybrid Plastics Inc. (Hattiesburg, MS, USA) were purchased. The MWCNTs obtained by chemical vapor deposition with purity of >98%, surface area of 250 m^2^/g, tube diameter in the range of 12 to 15 nm and 8 to 12 walls were provided by FutureCarbon GmbH (Bayreuth, Germany). Dehesive 940, crosslinker V24 and catalyst OL were purchased from Wacker Silicones GmbH (WackerChemie AG, München, Germany). All the organic solvents were used as received or were additionally distilled.

#### *Characterization*

Nuclear magnetic resonance (NMR) spectra of the compounds were recorded on Bruker AV-300 (Bruker Biospin GmbH, Karlsruhe, Germany) at 300 MHz using CDCl_3_. Fourier transform infrared spectroscopy (FTIR) was conducted using a Bruker Equinox 55 (Bruker Optics, Bremen, Germany). Thermogravimetric analysis (TGA) measurements were done using Netzsch TG209 F1 Iris (NETZSCH-Gerätebau GmbH,Selb, Germany), under constant argon flow of 20 mL/min at a constant heating rate of 20 °C/min. The transmission electron microscopy (TEM) characterization of MWCNTs was carried out in bright-field and energy filtered modesusing an FEI Tecnai G2 F20 (FEI Company, Eindhoven, The Netherlands) operated at 200 kV. Optical microscopy images were taken on Leica DMLM (Leica Microsystems GmbH, Wetzlar, Germany),and samples were analyzed under reflection mode. Scanning electron microscopy studies on the membranes were carried out using LEO 1550 VP from Zeiss (Carl Zeiss Inc., Oberkochen, Germany). The sample preparation for cross-section analysis was done under cryogenic conditions. The surface conductivity was analyzed with a four-point measurement equipment from Jandel, Linslade, UK. Gas permeances of CH_4_, N_2_, O_2_, CO_2_, and C_2_H_6_ were characterized at 23 °C using a constant volume variable pressure method facility.

### Experimental

#### *Synthesis of pyrene-POSS nanohybrid*

**Figure 1:**
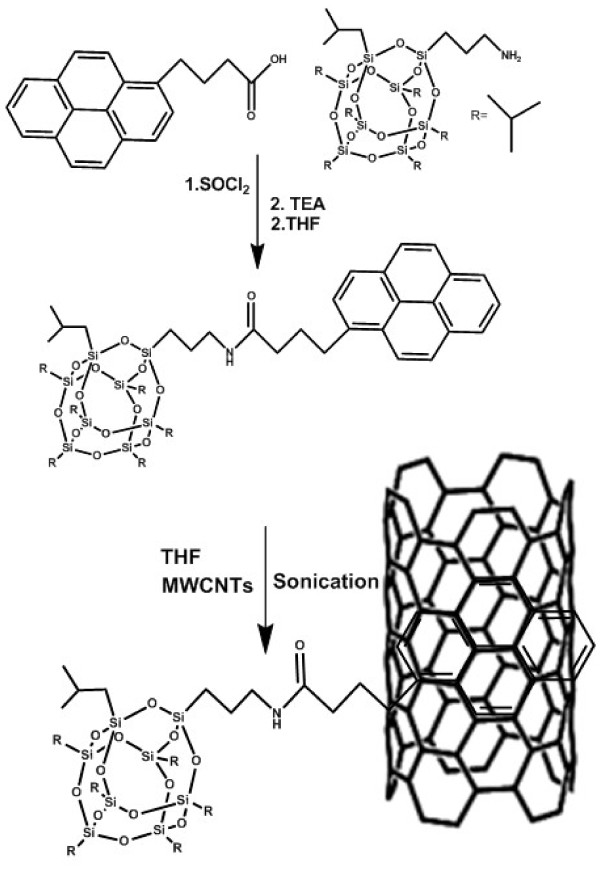
Reaction scheme for the preparation of pyrene-POSS and functionalization of MWCNTs with pyrene-POSS.

The reaction scheme for the synthesis of pyrene-POSS is shown in Figure 1. 1-pyrenebutyric acid (3.5 mmol) and an excess amount of thionyl chloride (68 mmol) were transferred to a 25-mL flask and refluxed at 65 °C for 2 h. After reflux, unreacted thionyl chloride was removed by distillation until the dry pyrenebutyryl chloride remained in the flask. The solid pyrenebutyryl chloride was dissolved in 5 mL anhydrous tetrahydrofuran (THF). APiB-POSS (3.5 mmol) and triethylamine (7.1 mmol) were dissolved in 10 mL anhydrous THF separately. The APiB-POSS solution was taken in an additional funnel and added dropwise to the pyrenebutyryl chloride solution below 0 °C. After complete addition of APiB-POSS, the reaction content was stirred for 20 h at ambient temperature. The reaction mixture was dissolved in diethyl ether and washed with water excessively. After washing with water, the organic layer was extracted and dried over sodium sulfate. Finally,diethyl ether was evaporated and the product was vacuum-dried at room temperature resulting in the product with 80% yield.

#### *Functionalization of MWCNTs*

Pyrene-POSS (300 mg) was dissolved in 50 mL THF, and dry MWCNTs were added to reach 1:1 weight ratio. The resultant mixture was sonicated for 5 h using Sonorex Super RK 255 H ultrasonic bath from Bandelin (Bandelin Electronic GmbH & Co. KG, Berlin, Germany), operated at a frequency of 35 KHz and an effective power of 160 W. The temperature was maintained at 20–25 °C during sonication. After sonication, MWCNTs were washed at least five times with THF to remove non-adsorbed pyrene-POSS followed by vacuum drying at room temperature for 24 h.

The functionalization of MWCNTs was also carried out at 1:0.2, 1:0.6, 1:2, and 1:3 weight ratios between MWCNTs and pyrene-POSS, respectively, to investigate the effect of the increased quantity of pyrene-POSS on its degree of adsorption on MWCNTs.

#### *Fabrication of nanocomposite membranes*

Appropriate amounts of pyrene-POSS-modified MWCNTs (PP-MWCNTs) were dispersed in 25 g toluene by sonication. Dehesive 940 was added to the PP-MWCNTs’ dispersion to maintain a 4 wt.% concentration of PDMS/PP-MWCNTs and 1, 2, 3, 4, and 5 wt.% loading of the effective MWCNTs with respect to PDMS. The mixture was stirred for 3 h to obtain a homogeneous dispersion, and crosslinker and catalyst were added to the solution followed by brief vigorous stirring to homogenize the system. The PDMS nanocomposite solution was dip-coated on top of a polyacrylonitrile (PAN) microporous membrane (average pore size 20 nm and 15% surface porosity); the solvent was evaporated at room temperature, and PDMS was cross-linked in an oven at 70 °C for 1 h.

## Results and discussion

Covalent functionalization of carbon nanotubes with APiB-POSS has been reported in literature for the case of MWCNTs dispersion in THF [20]. In the present study, we report the preparation of nanohybrid dispersant molecules based on APiB-POSS and pyrene for the physical surface modification of MWCNTs. Besides conserving the intrinsic properties of the MWCNTs, we aim to improve their dispersibility not only in a polar solvent like THF, but also in non-polar solvents like toluene and *n*-hexane. The improved dispersion of MWCNTs in non-polar solvents provides a way to fabricate PDMS nanocomposite films by solvent evaporation from a mixed solution of polymer and nanofiller.

### Characterization of pyrene-POSS

**Figure 2:**
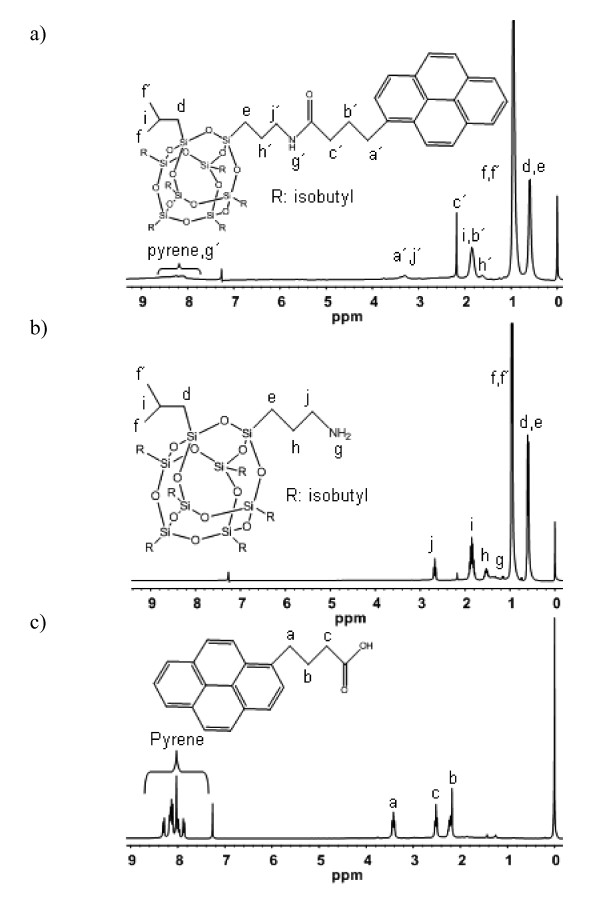
^**1**^**H-NMR spectra measured in CDCl**_**3**_**(a) pyrene-POSS, (b) APiB-POSS, and (c) 1-pyrenebutyric acid.**

**Figure 3:**
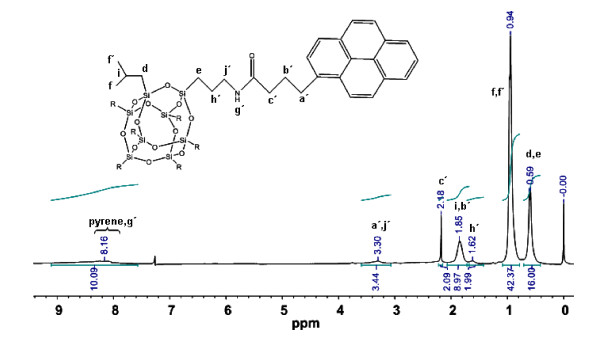
**Integration of**^**1**^ **H-NMR spectra of pyrene-POSS.**

The successful synthesis of pyrene-POSS hybrid is evident from ^1^ H-NMR measured in CDCl_3_. The amine protons of APiB-POSS (at 1.38 ppm in Figure 2) disappeared in pyrene-POSS after the amidation reaction. The appearance of the amide proton was confirmed by integrating the peak area of pyrene protons, whereby an increase in the peak area indicates the addition of one proton which is the amide proton (Figure 3). Figure 2 shows that after the amidation reaction, aliphatic chain protons of 1-pyrenebutyric acid i.e., a (3.42 ppm), b (2.18 ppm), and c (2.51 ppm) underwent shifts to a´ (3.30 ppm), b´ (1.85 ppm), and c´ (2.18 ppm), respectively, in pyrene-POSS. Also, h (1.53 ppm) and j (2.68 ppm) protons of APiB-POSS shifted to h´ (1.62 ppm) and j´ (3.30 ppm) in pyrene-POSS after reaction. These peak shifts clearly indicate the absence of unreacted 1-pyrenebutyric acid and APiB-POSS.

**Figure 4:**
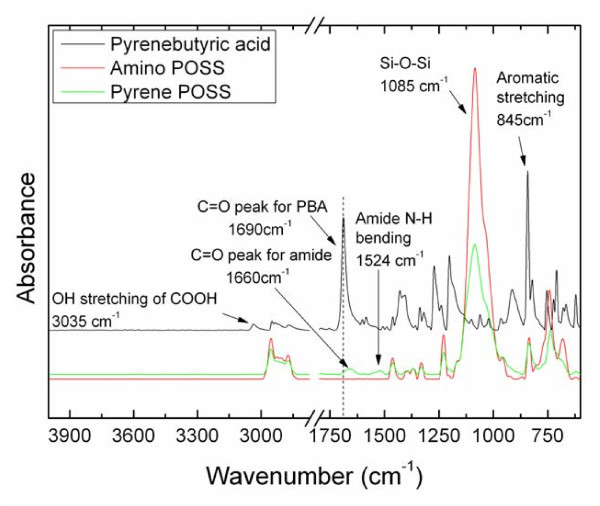
FTIR spectra of 1-pyrenebutyric acid, APiB-POSS, and pyrene-POSS.

The FTIR study also confirms the successful synthesis of pyrene-POSS (Figure 4). The spectrum of 1-pyrenebutyric acid shows the characteristic carbonyl peak at 1,690 cm^−1^. After amidation reaction this peak shifted to 1,660 cm^−1^ which is characteristic for the carbonyl peak of amide groups. The appearance of a peak at 1,524 cm^−1^ confirms the N-H bending of the amide group [25]. The OH stretching peak of 1-pyrenebutyric acid at 3,035 cm^−1^ disappeared in case of pyrene-POSS, which proves the complete conversion of carboxylic acid groups into amide groups. The typical Si-O-Si peak was observed in both APiB-POSS and pyrene-POSS at 1,085 cm^−1^.

### Dispersion of MWCNTs in solvents of different polarities

**Figure 5:**
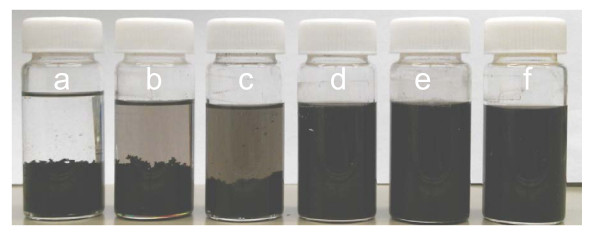
**MWCNTs dispersions without pyrene-POSS in bottles (a)*n*-hexane, (b) toluene, (c) THF and with pyrene-POSS (d)*n*-hexane, (e) toluene, (f) THF.**

Successful synthesis of pyrene-POSS could be confirmed by the analysis of MWCNTs dispersion stability in organic solvents. Dispersion analysis was carried out by dispersing purified MWCNTs in THF, toluene, and *n*-hexane. For the preparation of the dispersions, 5 mg of pyrene-POSS was dissolved in 25 mL of each solvent followed by the addition of MWCNTs leading to 1:1 weight ratio of MWCNTs and pyrene-POSS. Then, the resultant mixture was subjected to tip sonication (Sonoplus, Bandelin Electronic GmbH & Co. KG, Berlin, Germany) operated at 55 W for 15 min. The homogeneous dispersion of MWCNTs could be observed in all three investigated solvents which remained stable after storage for one week (Figure 5). However, the dispersions prepared in the absence of pyrene-POSS were unstable and showed a rapid sedimentation within 10 min. The sediments of MWCNTs in *n*-hexane and toluene can be observed easily, but MWCNTs sediments in THF were observed along with suspended MWCNTs in THF (Figure 5). In addition, the MWCNTs also underwent sedimentation when being dispersed in organic solvents in the presence of 1-pyrenebutyric acid and APiB-POSS, which are educts for the pyrene-POSS synthesis. This is a further strong indication of the dispersing property of this pyrene-POSS hybrid.

**Figure 6:**
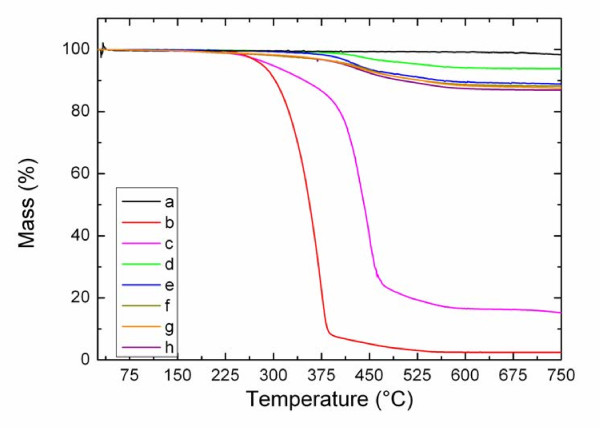
TGA of (a) purified MWCNTs, (b) ApiB-POSS, (c) pyrene-POSS, and (d to h) PP-MWCNTs using 1:0.2, 1:0.6, 1:1, 1:2, and 1:3 ratios of MWCNTs and pyrene-POSS, respectively.

TGA was used to confirm the physical adsorption of pyrene-POSS on MWCNTs. Figure 6 shows the TGA of pyrene-POSS, purified MWCNTs, and PP-MWCNTs. Purified MWCNTs did not show any mass loss until 700 °C. The decomposition of pyrene-POSS took place between 250 °C and 570 °C. The TGA of the samples with different ratios between MWCNTs and pyrene-POSS showed that the ratio has no significant influence on the adsorption degree of pyrene-POSS on the MWCNTs above weight ratio of 1:0.6. However, upon the investigated compositions, the 1:3 ratio between MWCNTs and pyrene-POSS showed the highest mass loss which may indicate the presence of free or weakly adsorbed pyrene-POSS (Table 1).

**Table 1:** TGA data for different ratios of MWCNTs and pyrene-POSS

MWCNTs/pyrene- POSS ratio^a^	Pyrene/ MWCNTs surface area ratio^b^	Mass loss (percent)^c^	Pyrene-POSS content (mmol g^−1^)^d^	Pyrene coverage (percent)^e^	Flat Pyrene-POSS coverage (percent)^e^	L-shaped pyrene-POSS coverage (percent)^e^
1:0.2	63:250	5.9	0.066	9.5	49	36
1:0.6	190:250	10.5	0.125	18	93	68
1:1	315:250	11.2	0.134	19	100	73
1:2	630:250	11.6	0.140	20	105	76
1:3	950:250	12.6	0.154	22	115	84

^a^Ratio of pyrene-POSS to purified MWCNT used for modification; ^b^surface area ratio by considering the area of pyrene unit as 60 Å^2^ in pyrene-POSS with respect to MWCNTs; ^c^mass loss of PP-MWCNTs according to TGA analysis (at 600 °C); ^d^quantity of pyrene-POSS immobilized on 1 g of purified MWCNTs; ^e^part of the MWCNTs surface covered by immobilized compound depending on the conformation.

**Figure 7:**
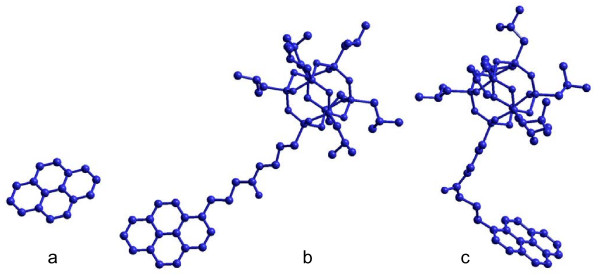
Conformations used for estimation of the MWCNTs surface (a) pyrene and pyrene-POSS in (b) flat and (c) L-shaped conformations.

The theoretical calculation of pyrene-POSS adsorption on the surface of MWCNTs (250 m^2^/g) was carried out by molecular modeling through measuring the surface area of pyrene and pyrene-POSS. The surface area of 60 Å^2^ as the projection of pyrene aligned to graphene sheet was calculated using AMBER molecular mechanic approach implemented in HyperChem version 8.0.3 molecular modeling software (Hypercube Inc., Gainesville, FL, USA) (Figure 7a). The projection area of two possible conformations of pyrene-POSS aligned to the same graphene surface was also calculated. One possible conformation can be represented as a pyrene-POSS molecule laying flat on a graphene sheet (Figure 7b) and gives the projection area of 310 Å^2^. Another considered conformation represents an “L-shaped” pyrene-POSS molecule (Figure 7c) formed by bending of the flexible chain connecting the pyrene and POSS moieties. The surface area of the projection of the “L-shaped” molecule to the graphene is 227 Å^2^. The graphene sheet consisting of 400 aromatic rings was created using HyperChem and was used for the surface area calculation, since the MWCNTs used in the current study have an average diameter of at least 12 nm which is at least six times larger than the biggest dimension of the pyrene-POSS molecule and can be approximated as a flat surface in comparison to the adsorbed molecules.

**Figure 8:**
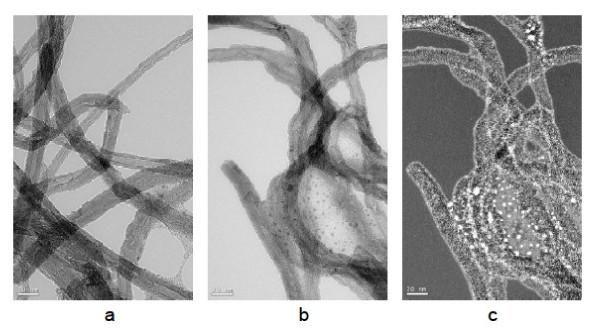
TEM images of (a) purified MWCNTs, (b) PP-MWCNTs, and (c) silicon mapping of PP-MWCNTs.

The part of the MWCNTs covered by each of the modeled molecules depending on the MWCNTs/pyrene-POSS ratio used for modification is presented in Table 1. It can be seen that the calculations done for all three molecules give very reasonable results. The most probable L-shaped conformation never exceeds 100% of the covered surface and thus seems as the most probable shape of the pyrene-POSS molecule adsorbed on the MWCNTs surface. The modeling results are in good agreement with the transmission electron microscopy results, distinctively showing pyrene-POSS adsorbed on the surface of the MWCNTs. The adsorbed pyrene-POSS molecules on MWCNTs are highlighted by silicon mapping where white spots represent places with the highest Si concentration (Figure 8c).

### Characterization of PDMS nanocomposite membranes

**Figure 9:**
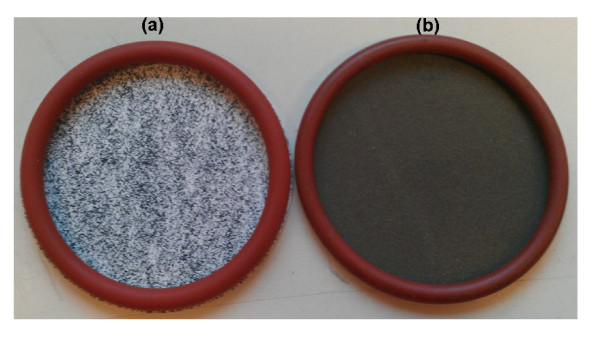
Photograph of PDMS membranes (a) 6 wt.% purified MWCNTs and (b) 3 wt.% PP-MWCNTs.

**Figure 10:**
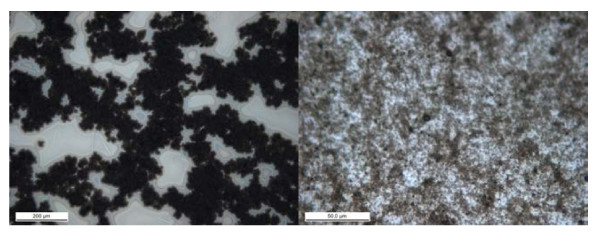
Optical micrographs of PDMS membranes with 6 wt.% purified MWCNTs, scale bar 200 μm (a) and 3 wt.% PP-MWCNTs, scale bar 50 μm (b).

The PDMS solution for membrane casting was comprised of toluene as solvent, Dehesive 940 as PDMS precursor, and PP-MWCNTs as fillers. The nanocomposite solution was cast on a dry PAN porous membrane using a dip-coating method. During the dip-coating process, the PDMS solution penetrates into the pores of the PAN substrate under the influence of capillary forces. After dip-coating the PDMS layer was cured at 70 °C for 1 h. As the PDMS coating was cast on the dry PAN porous support, some part of the solution penetrates into the pores, leaving PP-MWCNTs on the surface which stick in the remaining PDMS on the PAN surface. One advantage of using a dry porous support is the partial pore penetration of the solution which results in the increase of the effective percentage of PP-MWCNTs on the membrane surface which, in turn, reduces the required quantity of PP-MWCNTs to achieve percolation for electrical conductivity. The PDMS coating was fabricated using purified MWCNTs and PP-MWCNTs. A homogeneous black coating of PDMS was observed when PDMS was mixed with PP-MWCNTs. In contrary, the purified MWCNTs did not show homogeneous coating as black and white spots are clearly visible (Figure 9). It should be noted that the loading of purified MWCNTs in the digital image is twofold higher compared to PP-MWCNTs. The homogeneous black coating obtained using the PP-MWCNTs indicates the effectivity of the functionalization in terms of improvement of dispersion. The optical micrographs give a closer view of the PDMS coating (Figure 10) where the connected aggregates of purified MWCNTs are present along with MWCNT-free spaces (Figure 10 a). In contrary to these observations, the distribution of PP-MWCNTs appears quite homogeneous on the coating surface (Figure 10 b).

**Figure 11:**
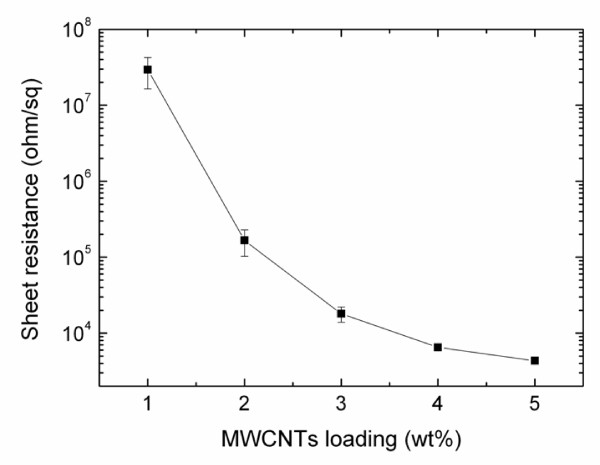
**Electrical sheet resistance (Ω/□)of PDMS membranes as a function of PP-MWCNTs loading.**

The sheet resistance of PDMS nanocomposite coatings was measured by a four-point probe technique. As expected, the sheet resistance decreases upon the increase in PP-MWCNTs content (Figure 11). The percolation threshold of PP-MWCNTs appears at approximately 2 wt.% loading where the sheet resistance becomes lower than 10^6^ Ω/□. The application of conductive coatings on porous substrates like foams might improve their ability to avoid electrostatic discharge [26]. In plastic housed spiral wound modules, electrostatic charges could be build up on the membranes surface by the friction of gas/vapor mixtures during the separation process reaching the limit of the breakthrough field strength and resulting in an electric discharge between membrane elements and causing severe membrane damage. The elimination of the static charge by a PDMS conductive coating may enhance the safety of the gas separation process in spiral wound membrane modules by dissipating/grounding the charges accumulated at the surface of the membranes.

**Figure 12:**
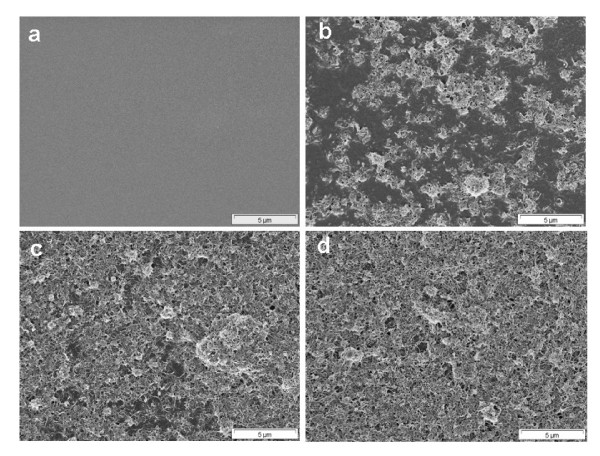
Surface morphology of PDMS membranes loaded with PP-MWCNTs (a) 0 wt.% (b) 1 wt.% (c) 3 wt.%, and (d) 5 wt.%; scale bar 5 μm.

**Figure 13:**
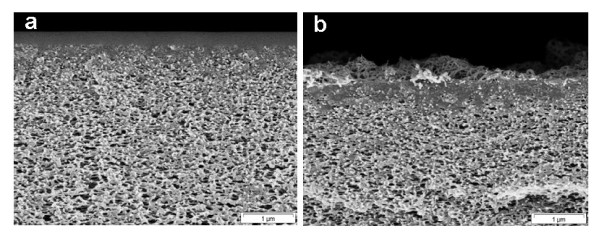
**Cross section of PDMS membranes (a) pure and(b) loaded with 3 wt.% PP-MWCNTs; scale bar 1 μm.**

Scanning electron microscopy (SEM) studies were carried out to analyze the surface and cross-sectional morphology of the nanocomposite membranes. The pure PDMS membrane has a smooth surface but it becomes rougher with the addition of PP-MWCNTs (Figure 12). Although the membrane surface looks homogeneously gray at 1 wt.% PP-MWCNTs loading by visual observation, in the SEM pictures, PP-MWCNTs agglomerates are visible on the surface along with PDMS patches. At 3 and 5 wt.% loading, PP-MWCNTs form dense carpets fixed with PDMS on the surface and in the pores. The PDMS layer with 5 wt.% PP-MWCNTs loading becomes unstable and can be scratched off by lightly rubbing the membrane surface. The cross-sectional view of pure PDMS and PDMS with 3 wt.% PP-MWCNTs is shown in Figure 13. It can be observed that the PDMS solution penetrates into the pores and covers the porous surface structure of the PAN membrane leading to a defect-free PDMS selective layer, whereas PP-MWCNTs stick at the surface in case of the nanocomposite membrane.

**Figure 14:**
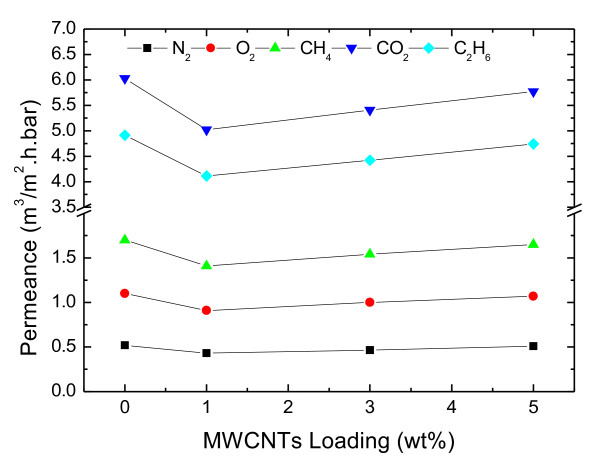
Gas permeance as a function of PP-MWCNTs loading.

**Figure 15:**
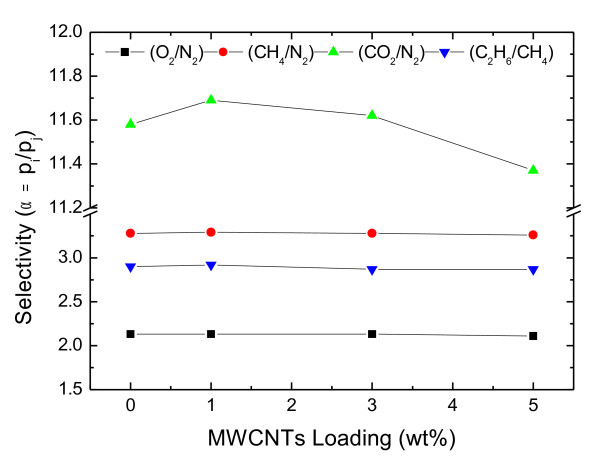
Selectivity of different gas pairs as a function of PP-MWCNTs loading.

The characterization of the defect-free PDMS selective layer was done by gas permeance measurements. The permeance of different gasses through PDMS nanocomposite membranes was measured by a “constant volume variable pressure” unit at 23 °C, the standard method to evaluate gas fluxes through composite membranes. Figure 14 depicts the permeance of the different gasses as a function of PP-MWCNTs loading. At 1 wt.% PP-MWCNTs content, a slight decrease in the permeance for all gasses is observable in comparison to a pure PDMS sample. With increasing PP-MWCNTs content above 1 wt.%, the permeance increases again but does not reach the value of the pure PDMS sample even at PP-MWCNTs content of 5 wt.%. The selectivity of the described gasses remained the same for composite membranes with pure PDMS layers and PDMS layers with incorporated PP-MWCNTs (Figure 15). The results are in accordance with the values reported in literature [27-29]. These values clearly indicate the formation of defect-free PDMS selective layers.

## Conclusions

Pyrene-POSS was synthesized via amidation reaction between 1-pyrenebutyric acid and aminopropyl*i*sobutyl POSS. The successful synthesis of pyrene-POSS hybrid is evident from NMR, FTIR, and MWCNTs dispersion analyses. TGA and TEM analyses proved the adsorption of pyrene-POSS nanohybrids on MWCNTs. Digital photographs and optical micrographs of PDMS nanocomposite membranes containing pyrene-POSS functionalized MWCNTs show homogeneously coated samples compared to samples containing purified MWCNTs. Defect-free conductive PDMS nanocomposite membranes were prepared which can reduce the probability of electrostatic discharges in gas separation applications. Moreover, the ability of pyrene-POSS to disperse MWCNTs in different solvents opens the way of dispersibility improvement of other carbon-based materials and provides the opportunity to fabricate polymer nanocomposites by solvent evaporation.

## Competing interests

The authors declare that they have no competing interests.

## Authors’ contributions

SM conducted the experiments and prepared the manuscript, JW contributed in membrane casting, CA conducted and interpreted the TEM measurements, SS carried out the modeling and helped in manuscript preparation, VF and VA supervised the study. All the authors read and approved the final manuscript.
